# Antiviral effects of *Scaptotrigona postica *propolis and their fractions

**DOI:** 10.1186/1753-6561-8-S4-P63

**Published:** 2014-10-01

**Authors:** Guilherme Rabelo Coelho, Karina de Senna Villar, Cristina Adelaide Figueiredo, Juliana Cuoco Badari, Rita Maria Zucatelli Mendonça, Maria Isabel Oliveira, Suely Pires Curti, Patrícia Evelin Silva Silva, Roberto Manoel Do Nascimento, Ronaldo Zucatelli Mendonça

**Affiliations:** 1 Instituto Butantan, Laboratório de Parasitologia, São Paulo, Brazil; 2 Instituto Adolfo Lutz, Laboratório de Virologia, São Paulo, Brazil; 3 Instituto Butantan, Laboratório de Virologia, São Paulo, Brazil; 4 Instituto Butantan, Laboratório de Ecologia e Evolução, São Paulo, Brazil

## Introduction

Studies about viral infections have a great importance in human and veterinary health, and the number of medications available to treatment these diseases is very reduced, making the search for antiviral molecules an important focus for scientific research. Propolis is a bee material manufactured by the mix of exudate of plants, saliva and bee wax. This product is used to seal the hive and involve dead invaders. Their chemical profile is very variable, and depending on the geographic origin and plant conditions of growth. The use of propolis (bee-glue) for various purposes has reports at before Christ. In Egypt, propolis was used in the preservation of bodies, performing a function of balsam, and its use persists to today in folk medicine to treat various pathologies, being widely used around the world. It is known that propolis of Apis Mellifera has compounds with antiviral activity on virus like Influenza A and B, Vaccinia virus, Hepatitis virus, HIV, Herpes virus, HIV and Poliovirus. In Brazil exists a subfamily of bees named Meliponinae, the stingless bees mixing the resins collected of plants with wax and ground, producing a different type of propolis, named geopropolis, but not all bees of this family produce this type of propolis, like Scaptotrigona postica. The biological activities of *Scaptotrigona postica *propolis remain unknown, the little information about this product is concentrated in antibacterial and anti fungi actions, but don't has related of antiviral action.

## Objectives

Purify, isolate, and characterize substances with antiviral activity of propolis from *Scaptotrigona postica*.

## Material and methods

The propolis collected in Barra do Corda, city of Maranhão state, was partitioned with hexane, ethyl acetate and a solution of water/methanol (1:1). The propolis crude, as well its purified fractions, were tested by viral titer reduction technique, and determination of viral mRNA against measles, picornavirus, influenza virus and rubella virus.

## Results and discussion

Experiments with the purified fraction led to a 64-fold reduction of picornavirus production, 32-fold reduction in influenza virus production and 8-fold reduction of measles virus. Assays using RT-PCR, to determine viral mRNA present in the treated and infected cells, also was performed. The purified antiviral fraction was able to reduce at 10^3 ^times the replication of rubella virus. At the moment, we are performed the optimization of the purification process.
